# Active pain coping is associated with the response in real-time fMRI neurofeedback during pain

**DOI:** 10.1007/s11682-016-9547-0

**Published:** 2016-04-12

**Authors:** Kirsten Emmert, Markus Breimhorst, Thomas Bauermann, Frank Birklein, Cora Rebhorn, Dimitri Van De Ville, Sven Haller

**Affiliations:** 1 Department of Radiology and Medical Informatics, CIBM, University Hospital Geneva, Gabrielle-Perret-Gentil 4, 1205 Geneva, Switzerland; 2 Medical Image Processing Laboratory, Institute of Bioengineering, Ecole Polytechnique Fédérale de Lausanne (EPFL), Lausanne, Switzerland; 3 Department of Neurology, University Medical Center of the Johannes Gutenberg-University Mainz, Mainz, Germany; 4 Institute of Neuroradiology, University Medical Center of the Johannes Gutenberg-University Mainz, Mainz, Germany; 5 Affidea Centre de Diagnostic Radiologique de Carouge CDRC, Geneva, Switzerland; 6 Faculty of Medicine of the University of Geneva, Geneva, Switzerland; 7 Department of Surgical Sciences, Radiology, Uppsala University, Uppsala, Sweden; 8 Department of Neuroradiology, University Hospital Freiburg, Freiburg im Breisgau, Germany

**Keywords:** Real-time fMRI, Neurofeedback, fMRI, Pain, Pain coping, CSQ

## Abstract

Real-time functional magnetic resonance imaging (rt-fMRI) neurofeedback is used as a tool to gain voluntary control of activity in various brain regions. Little emphasis has been put on the influence of cognitive and personality traits on neurofeedback efficacy and baseline activity. Here, we assessed the effect of individual pain coping on rt-fMRI neurofeedback during heat-induced pain. Twenty-eight healthy subjects completed the Coping Strategies Questionnaire (CSQ) prior to scanning. The first part of the fMRI experiment identified target regions using painful heat stimulation. Then, subjects were asked to down-regulate the pain target brain region during four neurofeedback runs with painful heat stimulation. Functional MRI analysis included correlation analysis between fMRI activation and pain ratings as well as CSQ ratings. At the behavioral level, the active pain coping (first principal component of CSQ) was correlated with pain ratings during neurofeedback. Concerning neuroimaging, pain sensitive regions were negatively correlated with pain coping. During neurofeedback, the pain coping was positively correlated with activation in the anterior cingulate cortex, prefrontal cortex, hippocampus and visual cortex. Thermode temperature was negatively correlated with anterior insula and dorsolateral prefrontal cortex activation. In conclusion, self-reported pain coping mechanisms and pain sensitivity are a source of variance during rt-fMRI neurofeedback possibly explaining variations in regulation success. In particular, active coping seems to be associated with successful pain regulation.

**Electronic supplementary material:**

The online version of this article (doi:10.1007/s11682-016-9547-0) contains supplementary material, which is available to authorized users.

## Introduction

Real-time functional magnetic resonance imaging (rt-fMRI) neurofeedback recently became a popular method to learn voluntary regulation of brain activity. As it is a rather new technique, publications have focused to date mostly on the technical feasibility and validity of the technique and its possible applications in different clinical fields such as chronic pain (deCharms et al. 2005), schizophrenia (Ruiz et al. 2013), tinnitus (Haller et al. 2010) and depression (Linden et al. 2012). Thus, mainly the neuroimaging results and behavioral outcome measures for the examined clinical populations were assessed. However, it is known that neurofeedback efficacy varies considerably between subjects (Johnston et al. 2011; Weiskopf et al. 2003; Emmert et al. 2014), yet the origin of this inter-individual variability remains poorly investigated.

Here, we looked to find domain-specific behavioral factors that influence neurofeedback using previously published neurofeedback data regulating pain sensitive areas (Emmert et al. 2014). Brain areas involved in pain perception include the primary and the secondary sensory cortex and the posterior insula (Peyron et al. 2000; Apkarian et al. 2005; Tracey 2005). Areas involved in pain arousal and emotion, pain consequences and pain modulation include the anterior cingulate cortex (ACC), the anterior insula (AIC), prefrontal cortical areas and subcortical areas (including the basal ganglia and the thalamus) (Apkarian et al. 2005; Friebel et al. 2011). In addition, brainstem structures including the periaqueductal gray (PAG) and the ventral tegmental area are also implicated in perception and modulation of pain by controlling the gain of pain transmission from the spinal cord (Apkarian 2008). It has been shown that pain perception and processing is influenced by a variety of psychological factors. For example, this is evident when looking at the placebo/ nocebo effect that influences pain related brain activation (Bingel 2010; Kong et al. 2008; Lidstone and Stoessl 2007). Two recent meta-analyses on placebo neuroimaging studies showed that expected pain reduction is accompanied by a reduction in dorsal ACC and MCC, insula, thalamus, amygdala, striatum, superior temporal and precentral gyri and lateral prefrontal cortex activation, as well as an increase in activation in the dorsolateral and ventromedial prefrontal cortex, the left inferior parietal lobule and postcentral gyrus, the rostral ACC, the midbrain around the PAG, the left anterior insula, and the striatum (Atlas and Wager 2014; Amanzio et al. 2013).

There are attempts to use the link between cognition and brain activation to alter pain processing through different behavioral strategies including distraction-based techniques, cognitive behavioral therapy and mental imagery (Flor 2014; Jensen et al. 2012). The ACC and the AIC seem to be of particular importance for the perception of pain intensity and affect (Favilla et al. 2014), especially in neurofeedback studies (deCharms et al. 2005). Previous neurofeedback showed successful regulation of the AIC in healthy participants (Lawrence et al. 2013; Caria et al. 2007), obese participants (Frank et al. 2012) and in schizophrenic patients (Ruiz et al. 2013) although up-regulation seems to be easier than down-regulation (Veit et al. 2012). The ACC was mainly regulated in the context of pain studies. A previous pilot study in patients with chronic pain (deCharms et al. 2005) found that anterior cingulate cortex (ACC) regulation using rt-fMRI neurofeedback resulted in a decrease of pain intensity. Further research with healthy participants confirmed that down-regulation of the ACC is possible (Rance et al. 2014; Emmert et al. 2014). However, up-regulation was not successful (Rance et al. 2014) and researchers found that effects of pain regulation through neurofeedback vary between subjects (DeCharms 2012).

In our previous study (Emmert et al. 2014), we compared neurofeedback efficacy during pain using either the AIC or the ACC as the target region. Even though our results suggested that the majority of both groups were able to regulate the target area, the effect size varied substantially between subjects, leading to the hypothesis that there is an unexplained variability during neurofeedback. Concerning pain neurofeedback studies, these differences might be related to how subjects cope with pain in general.

Individual pain coping behavior can be assessed by the Coping Strategies Questionnaire (CSQ) (Rosenstiel and Keefe 1983), a self-reporting questionnaire. The CSQ has been repeatedly applied to healthy subjects in experimental pain studies (Hastie et al. 2004; Lefebvre et al. 1995; Lester et al. 1996; Campbell et al. 2005; Kashikar-Zuck et al. 1997). The active score of the CSQ is of particular interest for brain regulation during pain, as it was shown to predict perceived control over pain (in particular the sub-scale self-statement) (Haythornthwaite et al. 1998) and self efficacy (Keefe et al. 1997). Therefore, we use the CSQ as a tool to investigate the association between individual coping behavior and brain activity during neurofeedback as a source of inter-individual variability in neurofeedback pain paradigms.

## Material and methods

### Participants

Twenty-eight healthy subjects (mean age: 27.5 ± 2.3 years, 14 male, 14 female) gave written informed consent to participate in this study that was approved by the local ethics committee of the Rhineland Palatinate medical association in Mainz, Germany. Participants were randomly assigned to two groups of 14 participants each, including seven men and women per group (AIC-Group: 27.6 years ± 2.1, ACC-Group: 27.4 ± 2.6 years). The left anterior insula (lAIC) served as a target region for feedback in the first group while the second group received feedback from the ACC. Exclusion criteria were acute or chronic pain, pregnancy, severe neurological or internal disorders, intake of painkillers and contraindications for MR-measurements. All participants received financial compensation for the study.

### Assessment of pain coping behavior

**Fig. 1:**
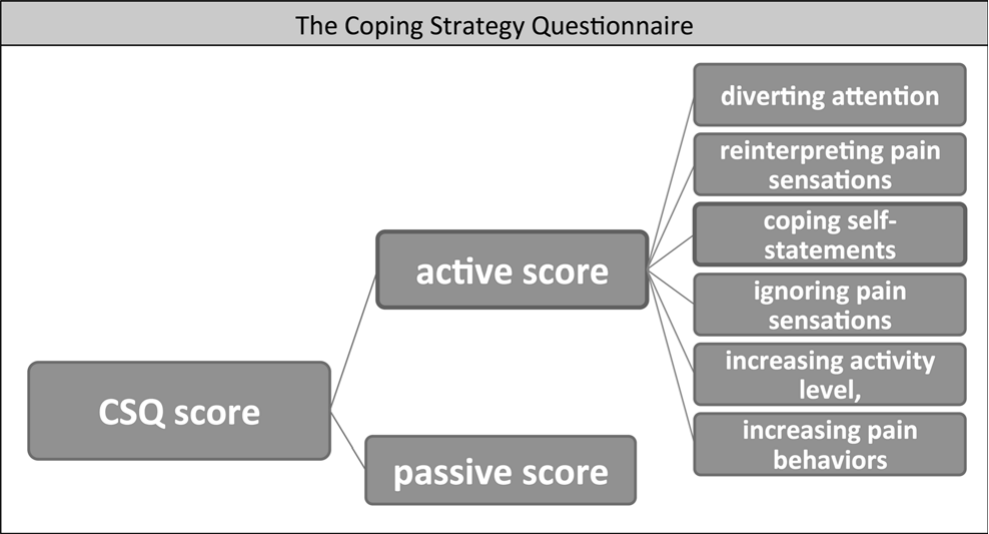
Structure of the Coping Strategy Questionnaire (CSQ) assessing personal pain coping

Before undergoing the experiment, all subjects completed the CSQ (for an overview of the CSQ structure see Fig. 1). The score for active coping consists of six sub-scores (diverting attention, reinterpreting pain sensations, coping self-statements, ignoring pain sensations, increasing activity level, increasing pain behaviors) and is the main behavioral outcome parameter assessing coping strategies. Each sub-score is calculated from ratings of six strategies each (randomly distributed in the questionnaire) and subjects used a 7-point Likert scale ranging from 0 (“never do that”) and 6 (“always do this”) to rate how often they use or would use each strategy to cope with pain. As an example, the self-statement score is calculated from the six items listed in list 1.

List 1: Items of the CSQ self-statement score (extracted from Verra et al. 2006; Rodriguez Franco et al. 2004)

**Table:** 

Subjects are asked to rate from 0 (never) to 6 (always) what they do when in pain. 1.) I tell myself to be brave and to carry on despite the pain. 2.) I tell myself I can’t let the pain stand in the way of what I have to do. 3.) I see it as a challenge and don’t let it bother me. 4.) I tell myself that I can overcome the pain. 5.) No matter how bad it gets, I know I can handle it. 6.) I keep on going although it hurts.	

### Real-time experiment

**Fig. 2:**
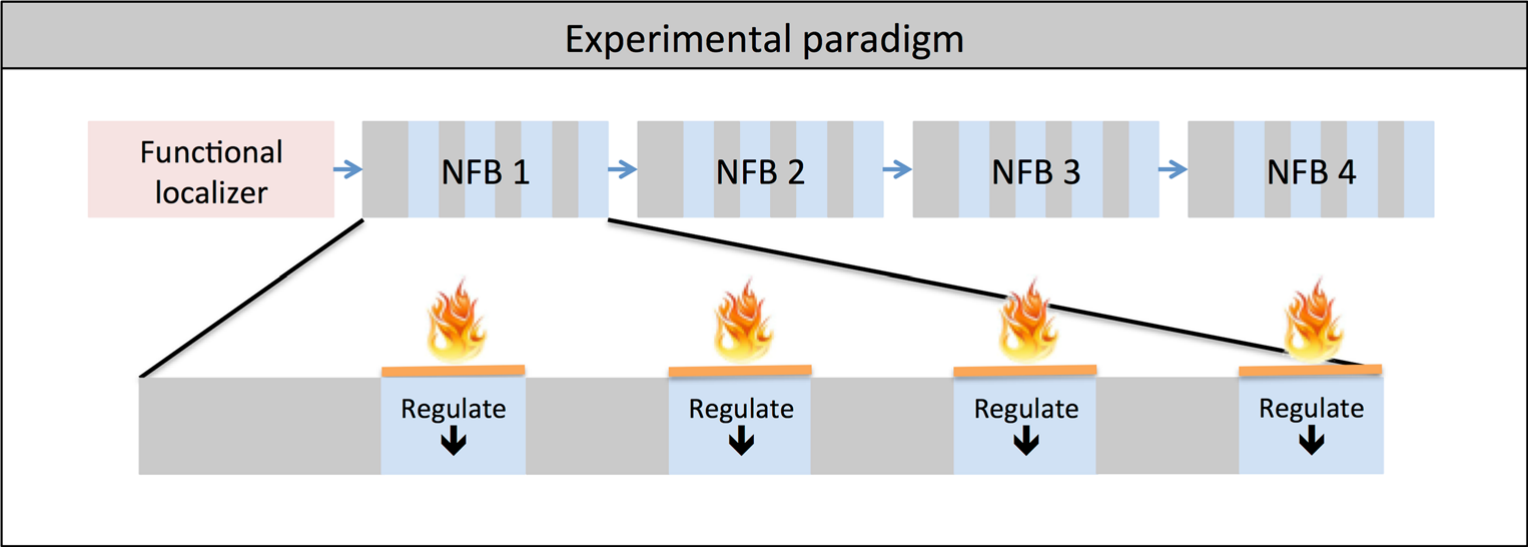
Experimental design: each of the four neurofeedback runs (NFB) consists of four regulation blocks of 30 s each with pain stimulation

For a detailed description of the paradigm the reader is referred to the initial description of this data set (Emmert et al. 2014). Prior to the neurofeedback part of the experiment, a functional localizer ran with an ON-OFF block design of eight blocks alternating between continuous painful heat stimulation for 30 s and rest for 30 s each. This was carried out to identify each individual’s target region. Thereafter, the main experiment of four identical neurofeedback runs was conducted. Each run consisted of a block design of four rest and regulation blocks (30 s each) proceeded by 15 s of initial rest before the first block (see Fig. 2). Online data analysis was performed using TurboBrainVoyager (Brain Innovation, Maastricht, The Netherlands, Version 2.8). The target region was chosen based on significant activation within the lAIC/ACC during the functional localizer. During regulation phases, the same pain stimulation as during the localizer was undertaken. In addition, subjects were requested to decrease the target region activation represented by a yellow line. The background color of the yellow line indicated to either keep the yellow line constant (black = rest blocks, no heat pain) or to decrease the amplitude of the yellow line (blue = down-regulation, heat pain). Subjects could freely choose their own mental strategy to decrease target region activation. They were not informed about any link between their task and their pain experience. Employed strategies are summarized in the supplementary Table 1.

### Pain stimulation and rating

Pain stimulation was performed using an MR compatible thermode (TSA 2001, Medoc Ltd, Ramat Yishai, Israel) placed on the middle of the right volar forearm. Initially, the thermode temperature was adjusted for each participant to elicit a subjective pain intensity of 7 out of 10 on a numeric rating scale (NRS). In this way, subjective pain was normalized so that pain rating differences towards the end of the experiment would not be caused by differences in pain sensitivity but the experiment itself. The thermode temperature was recorded for 26 out of the 28 subjects. This temperature for pain stimulation remained constant throughout the experiment. Pain ratings were obtained after each run (including functional localizer) using a 11-point NRS ranging from 0 (not painful) to 10 (most painful). The success of the neurofeedback was determined based on whether the pain rating decreased after neurofeedback (=success) or not.

### fMRI data acquisition

Neuroimaging was performed on a 3 T MRI Scanner (Siemens Tim Trio, Erlangen, Germany) with a 32-channel head-coil. Functional data acquisition used an echo-planar imaging sequence (EPI, TR = 1500 ms, TE = 30 ms, matrix size 64 × 64, 24 slices, slice thickness 3 mm without gap). Additionally, a high-resolution T1-weighted anatomical scan (magnetization prepared rapid gradient echo (MPRAGE), 1 mm isotropic) was used for later co-registration with the EPI images.

### Statistical analysis of pain ratings, thermode temperature and CSQ scores

Statistical testing for correlation between thermode temperature, pain ratings and the CSQ measures was carried out in MATLAB 2012b (The MathWorks, Inc., Natick, USA) using Spearman’s Correlation (two-sided). Due to the strong inter-dependencies of the six active sub-scales of the CSQ (diverting attention, reinterpreting pain sensations, coping self-statements, ignoring pain sensations, increasing activity level, increasing pain behaviors), Bonferroni correction would be too conservative to apply (Abdi 2007). Therefore, we undertook a principal component analysis for all subjects and all 6 active score sub-scales using single value decomposition to identify the first principal component that best represents the participant data of the six active CSQ sub-scales. This measure has the advantage of using the structure of the questionnaire (division into six sub-scales) as well as all sub-scales to a varying degree.

We then checked for correlation between this first component and pain ratings as well as thermode temperature.

### Post-hoc GLM activation correlation with behavioral measures

Off-line analysis was performed with FSL 5.0 (FMRIB Analysis Group, University of Oxford, UK). Functional data was spatially realigned, normalized and smoothed (FWHM = 5 mm kernel) in a first step.

Next, first level neuroimaging results were obtained by fitting a standard GLM regressor to the pain stimulation and neurofeedback blocks (block design described under “Assessment of pain coping behavior section”, for details on the main effect of neurofeedback please see Emmert et al. (2014)).

Finally, a voxel-wise regression analysis between the behavioral scores (PC1, pain rating and pain rating change between localizer and neurofeedback runs) and the imaging data (using the contrast of parameter estimates (COPE) files of the first level analysis) was performed using a mixed-effects GLM. The main regressor was the demeaned and normalized (values between −1 and 1) score of interest. To exclude the possibility that group-specific differences drive the effect we added non-explanatory co-regressors that model the neurofeedback group (AIC versus ACC target region).

For the fMRI analysis, voxels with a z-score above 2.3 within clusters that exceeded a multiple-comparison corrected significance threshold of *p* < 0.05 were considered significant.

## Results

### Principal component analysis of the active sub-scores

The principle component analysis (PCA) resulted in a first principal component (PC 1) with only positive weights, indicating that all six sub-scores positively contribute to this component (see Table 1). In particular, these weights indicate how different subscales explain the inter-subject variability (see Table 1). The sub-scores “diverting attention”, “ignoring pain sensation” and “increasing activity level” are most important. Overall, PC 1 is able to explain the majority of the variance (58.57 %).

**Table 1:** Weights of all CSQ active sub-scores for PC 1

Sub-score	Weight (U)
Diverting attention	0.5318
Reinterpreting pain sensations	0.1900
Coping self-statements	0.3361
Ignoring pain sensations	0.5377
Increasing activity level	0.4906
Increasing pain behaviors	0.1955

### Behavioral data: correlation of pain ratings, thermode temperature and CSQ scores

There were no significant differences in pain ratings and CSQ scores between the two groups with different NFB target region. Therefore, the analyses in this paper were conducted for all 28 NFB participants together, independent of the targeted ROI (AIC/ACC).

There was no significant correlation between baseline pain rating (after functional localizer) and the first PC. However, the thermode temperature (assessed in 26 out of the 28 subjects) was positively correlated with the localizer pain rating (*R* = 0.404, *p* < 0.05).

**Fig. 3:**
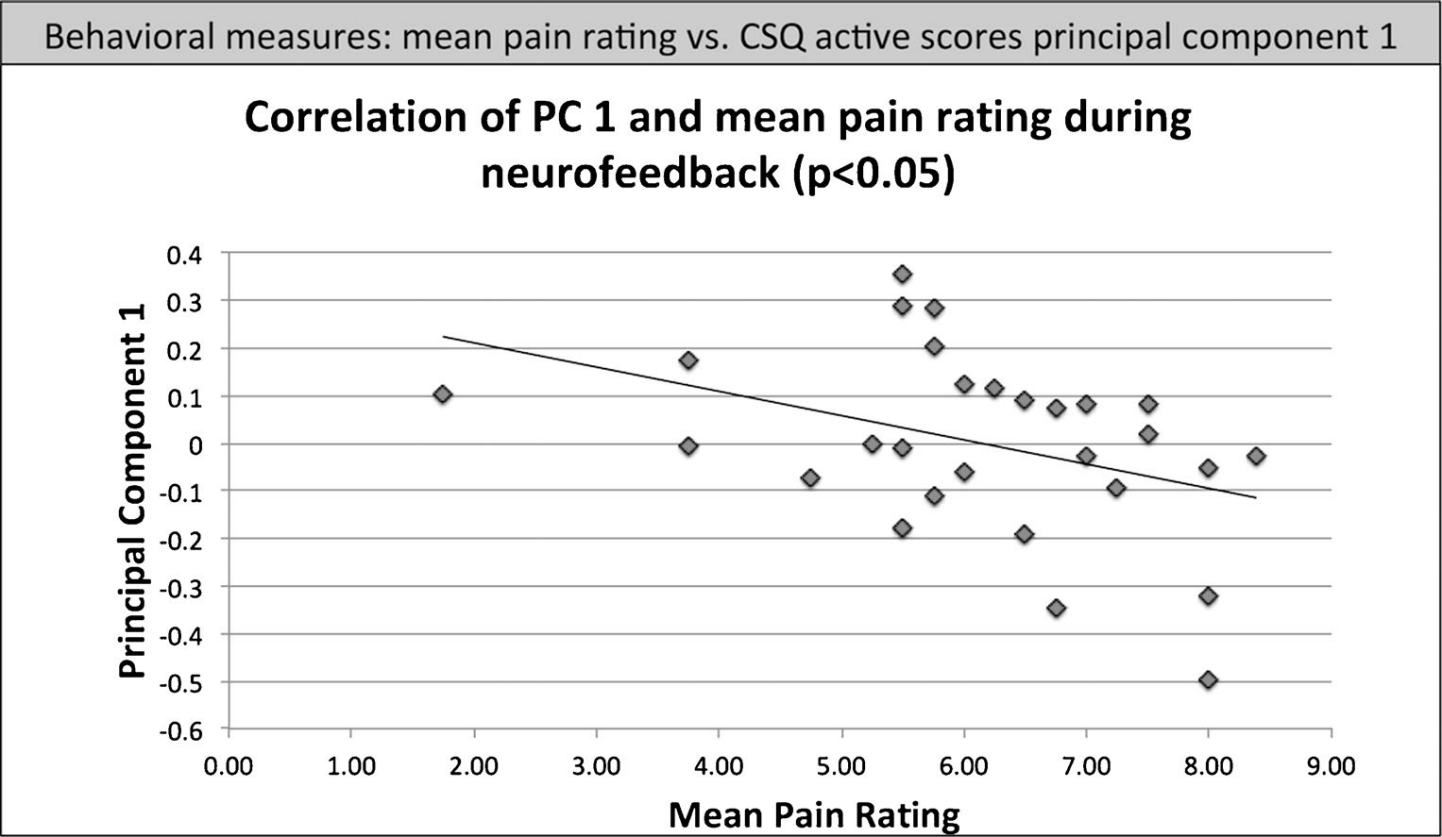
Pearson correlation of the mean pain rating during neurofeedback with PC1 (Rho = −0.393, *p* < 0.05)

The CSQ active first PC and the mean pain rating during neurofeedback runs (average of all 4 neurofeedback runs) yielded a significant (Rho = −0.393, *p* < 0.05, see Fig. 3) negative correlation: participants with a lower first PC had higher pain ratings.

### Pain stimulation: correlation of BOLD responses during the functional localizer run with CSQ scores

**Fig. 4:**
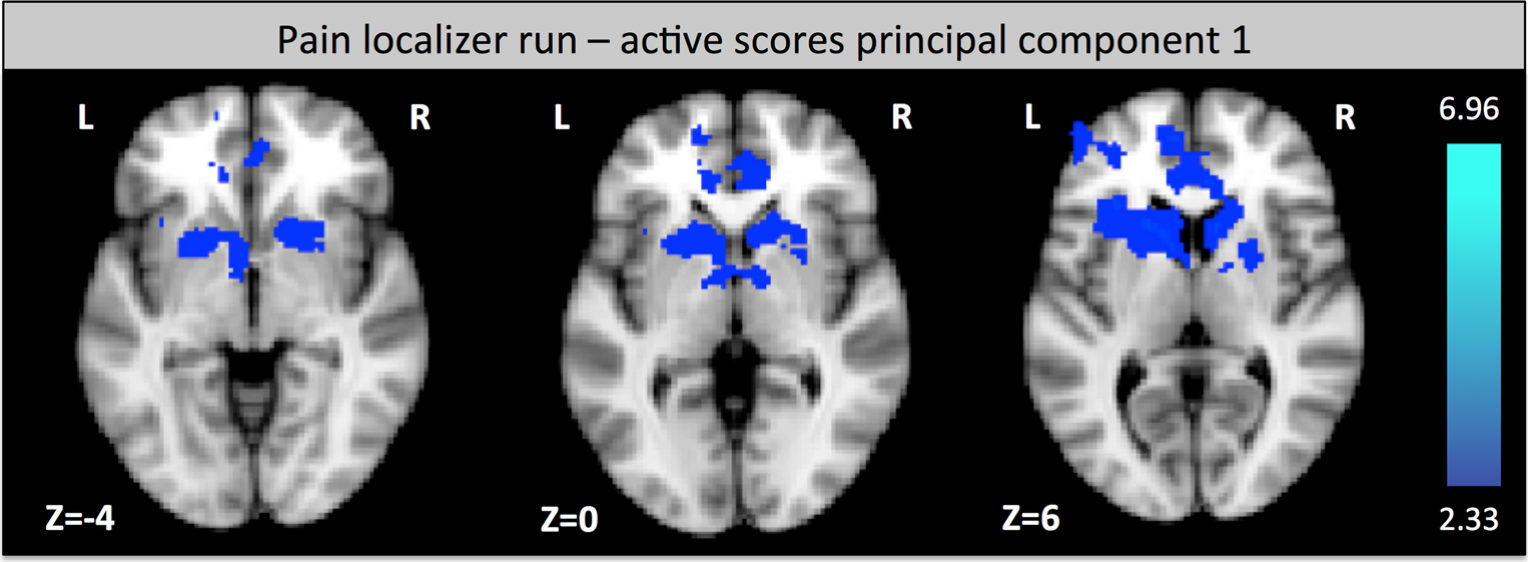
Brain activation correlation during the functional localizer: activation that is negatively correlated to PC 1 (active coping) during the functional localizer run (z-score > 2.3, cluster thresholding using *p* < 0.05)

During the functional localizer run, the first PC was negatively correlated with activation in the caudate nucleus and other neighboring parts of the striatum, the ACC and the lAIC (see Fig. 4). There is no positive correlation of the PC1 with brain activation.

There was no correlation between the thermode temperature and brain activity during the localizer run.

### Pain perception during neurofeedback: correlation of BOLD responses during neurofeedback with thermode temperature

**Fig. 5:**
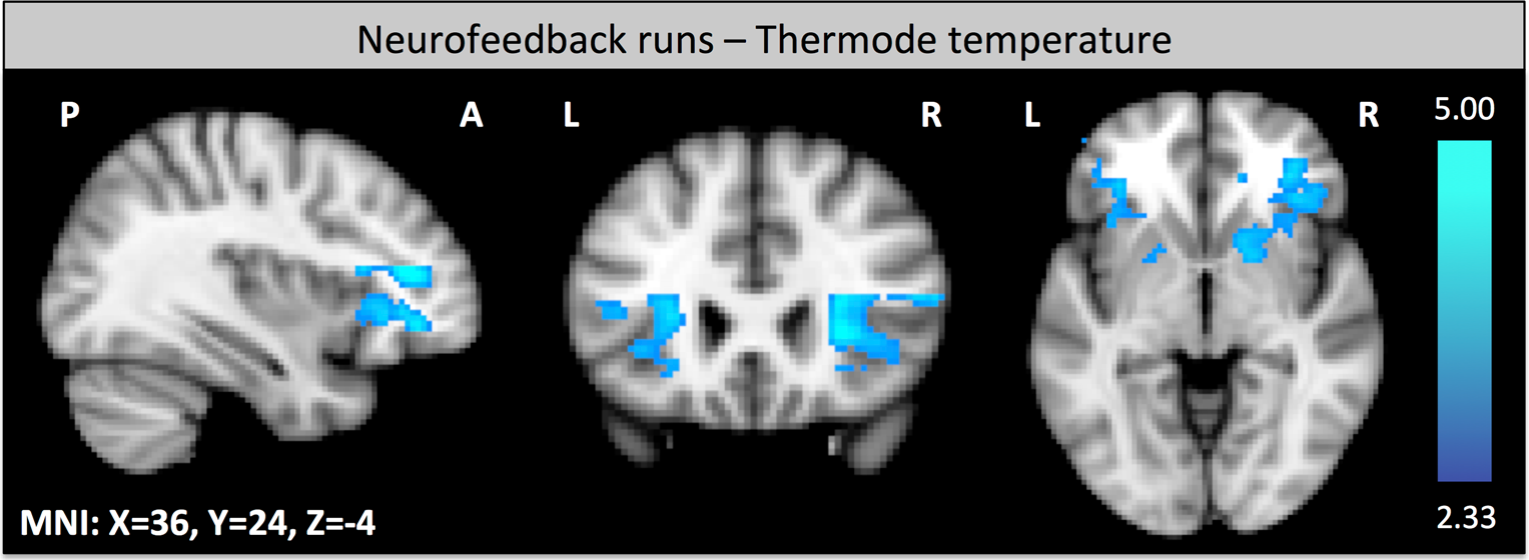
Brain activation correlation during the neurofeedback task: regions that are negatively correlated with the thermode temperature during neurofeedback runs (z-score > 2.3, cluster thresholding using *p* < 0.05)

Lower thermode temperature for the neurofeedback experiment was correlated with increased activity in the anterior insula and the dorsolateral prefrontal cortex (dlPFC, Brodman area 46) during neurofeedback runs (Fig. 5).

### Pain perception during neurofeedback: correlation of BOLD responses during neurofeedback with CSQ scores

**Fig. 6:**
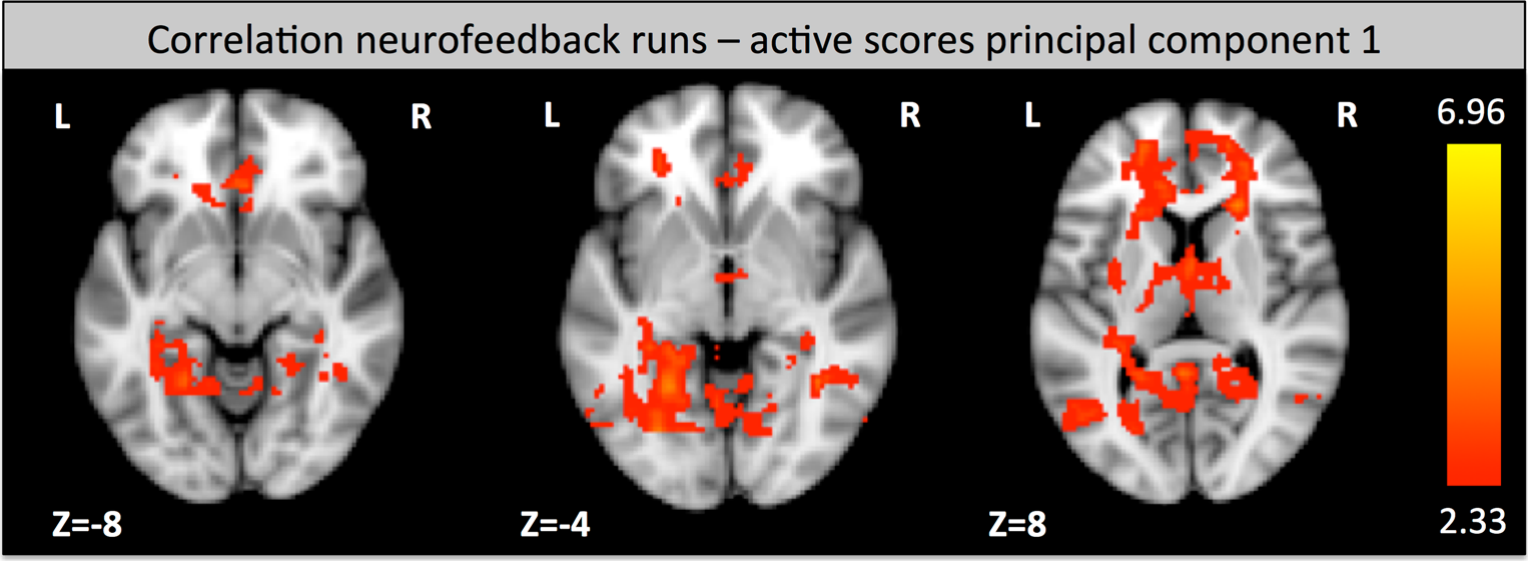
Brain activation correlation during the neurofeedback task: regions that show a positively correlated activation with PC 1 (active coping) during neurofeedback runs (z-score > 2.3, cluster thresholding using *p* < 0.05)

When looking at all the neurofeedback runs together, the active scores PC 1 were positively correlated with activation during neurofeedback in the ACC, prefrontal areas (Brodmann areas 9,10) and a small medial part of the left insula. In addition, there was a larger occipital activation, that was more extended on the left side stretching from the hippocampus to parts of the parahippocampal, occipital fusiform (including the peak voxel at −26 −76 −2 (MNI coordinates) with a z-score of 5.03) and lingual gyrus (Brodmann area 19), encompasing part of the cuneus (Brodmann area 18) and the thalamus (see Fig. 6). No negative correlations were found.

## Discussion

Personal pain coping capacity, specifically active coping, was associated with heat pain perception and the ability to influence pain processing with the help of real-time fMRI neurofeedback. During baseline pain, the first principle component of CSQ active sub-scores was associated with deactivation in striatum, ACC and lAIC. During neurofeedback, the PC 1 negatively correlated with the mean pain rating during neurofeedback. In addition, a high PC1 was associated with an increased activation in several brain areas including the ACC, the thalamus and visual areas during neurofeedback.

PCA was successfully used to reduce the dimensonality of the CSQ data, similar to another study looking at CSQ measures in patients with chronic back pain (Woby et al. 2005). Similarly, we excluded the passive measures of the CSQ, including the catastrophizing score, from the coping style analysis, as it does not “represent an effortful response to obtain support or assistance from others” (Woby et al. 2005, page 101). However, while Woby et al. looked at the interaction of catastrophizing and coping habits, we here used the first PC as a summarizing measure of active pain coping. We looked for correlation of this measure with pain rating and brain activity during neurofeedback. Our results show that active coping styles are associated with the success in neurofeedback; i.e., a smaller pain rating compared with participants with a lower PC 1 (as all weights of the PC 1 were positive). This explains the mixed response of subjects to neurofeedback with some showing successful regulation while others did not control their target region activity at all. Therefore, cognitive and personality traits, in particular those related to the regulated area, should be assessed before neurofeedback to preselect those subjects that are more likely to succeed.

### Behavioral data: correlation of coping activity, thermode temperature and pain rating

At the behavioral level, we assessed the effect of individual pain coping ability on pain rating during heat pain stimulation and real-time fMRI neurofeedback. We found no significant interaction of the active scores PC 1 and behavior during the baseline pain perception run. This result was expected as the pain stimulus (temperature of thermode) was individually adjusted for each subject to elicit a constant pain intensity (7 out of 10 on a NRS) prior to the localizer run and the participants were not trying to control pain. However, we found a positive correlation of the thermode temperature and baseline pain rating. This is not surprising, as higher thermode temperature should elicit more pain.

The pain during neurofeedback manipulation was negatively correlated to the CSQ active PC 1, indicating that active pain coping may influence pain perception during pain region rt-fMRI regulation.

### Correlation of neuroimaging and coping activity during pain stimulation without feedback

In a first step, we assessed brain activation during the functional pain localizer run without neurofeedback. Note that the pain stimulation paradigm was individually adjusted to evoke an individual pain response of 7 out of 10 on a NRS. This means that the subjective pain perception was the same for all subjects in the beginning of the experiment, whereas the actual absolute temperature may have varied between participants.

Despite the fact that the pain stimulation was adjusted to evoke the same degree of subjective pain, participants with a lower degree of active coping had increased activation in the striatum, especially the caudate nucleus, the ACC and the lAIC. This might indicate that pain processing is different in participants that are used to cope actively with pain. This view is supported by a study suggesting that intended pain suppression decreases ACC and caudate nucleus activation (Freund et al. 2007). Furthermore, it has been shown that the use of repeated positive self-statement can increase the pain sensitivity range, i.e. the difference between pain tolerance and threshold (Roditi et al. 2009). Conversely, catastrophizing self-statements sensitized for pain perception (Ruscheweyh et al. 2013). The decreased activity for actively coping participants might be accompanied by an increase in cortisol release, at least for women (Bento et al. 2010).

The fact that brain activation is different depending on active pain coping, even though the subjective pain perception is at the same level, indicates that active coping seems to be associated with the use of different resources during pain. This suggests that there might be a substantial individual variation of how pain is processed depending on the coping habits. A study by Roditi et al. (Roditi et al. 2009) found that the pain threshold remained stable while the pain tolerance (i.e. the time subjects can endure pain) is enhanced in subjects with a higher positive self-statement score. Our results indicate that a less negative/unpleasant perception of pain, indicated by a decrease of activity in pain-interpretation related areas, might be present in actively coping participants in the absence of differences in pain strength. The absence of behavioral effects in the presence of neuroimaging effects can be explained by the fact that pain perception at the behavioral level is influenced by many factors including fatigue, arousal and attention. Neuroimaging data is more directly able to assess subtle changes, especially with small sample sizes, as they are less prone to strong variation depending on these factors. This phenomenon has been observed in various neuroimaging studies, especially when expected effect sizes were low (e.g. Haller et al. 2013; Johnston et al. 2011; Weiskopf et al. 2003).

We found a significant correlation between activation of brain regions associated with pain arousal, emotional processing and modulation and individual active pain coping. Previous neuroimaging studies focused on a passive sub-scale of the CSQ questionnaire, namely the catastrophizing scale, and found that an increased catastrophizing score is associated with a high response in areas responsible for different aspects of pain (e.g., ACC, claustrum, medial frontal cortex, cerebellum) and motor control (Gracely et al. 2004).

High acceptance scores and low denial scores on a different coping questionnaire were shown to be related to ventrolateral prefrontal cortex activation (Salomons et al. 2007). In contrast to this study, we did not find any positive correlation between brain activation and coping scores. This discrepancy could be caused by the difference of focus of the two different coping questionnaires (pain acceptance versus active coping).

### Pain perception, thermode temperature and brain activation during neurofeedback

Thermode temperature (i.e. the intensity of the heat pain stimulus to yield pain rating of 7) was negatively correlated with the activity in the anterior insula and the dorsolateral prefrontal cortex (dlPFC, Brodman area 46) during neurofeedback runs. These results suggests that subjects with a higher pain sensitivity have an increased activity in pain related brain areas during neurofeedback. This explains why these subjects also show a smaller decrease in pain rating in comparison to the subjects with a lower pain sensitivity.

We also looked at the relation of active CSQ scores to neuroimaging data obtained during all neurofeedback runs. Active coping (high PC 1) was positively correlated with activation of occipital regions involved in vision, especially movement processing, ACC, prefrontal areas, left hippocampus and thalamus activation. One interpretation of the occipital activation is that participants with strong active coping used increasingly vivid mental imagery (Kosslyn et al. 2001) during neurofeedback. ACC and prefrontal involvement might be explained by a conscious effort to suppress pain. In line with this hypothesis, it has been shown that functional connectivity of the prefrontal cortex with the ACC and insula positively correlates with pain measures (Fomberstein et al. 2013). In rats, it has even been demonstrated that prefrontal cortex stimulation induces analgesia (Hardy 1985). Of note, the ACC is part of the pain network contributing to the processing of painful stimuli and part of the brain regulation network (Lee et al. 2012; Ninaus et al. 2013). It seems that among these conflicting processes an increased amount of self-regulation (associated with more active coping) leads to ACC hyperactivity even though pain perception is decreased.

Hippocampus involvement might reflect memory processes, possibly related to mental imagery as a neurofeedback tactic. In addition, thalamic activation might reflect altered somatosensory processing of pain or increased alertness due to more conscious effort exerted during the neurofeedback process for participants with stronger active coping. In total, active pain coping is associated with brain activation during neurofeedback, possibly reflecting a more vivid and dedicated regulation strategy.

### Does active coping increase the success of rt-fMRI neurofeedback?

We showed that active coping is positively correlated with regional brain activation during neurofeedback. The negative correlation of pain ratings with active coping PC 1 during neurofeedback runs indicates successful target brain region regulation as pain stimuli were normalized before the start of the experiment. This result is compatible with previous studies showing that positive self-statement predicts self efficacy (Keefe et al. 1997) and perceived control over pain (Haythornthwaite et al. 1998). In summary, active pain coping is associated with success in regulating brain activity.

## Limitations

A limitation of this study was the relative small sample size (*n* = 28) used. In addition, further studies are needed to determine whether these results can be generalized to neurofeedback in other domains; i.e., if active coping influences regulation success in general or if this is a specific effect in the domain of pain perception neurofeedback. Moreover, the current study used two different feedback sources (either AIC or ACC), therefore, the sample might be more heterogeneous than studies using only one feedback source for all subjects.

It should be noted that in this study, we are not able to differentiate between the pain regulation abilities independent of neurofeedback, as regulation without feedback was not tested beforehand. Therefore, the pain reduction cannot be attributed unequivocally to neurofeedback training alone. Similarly, we do not take learning mechanisms into account in this study, as the course of neurofeedback learning varies greatly between subjects and no specific model of learning has been shown to hold true for neurofeedback learning yet. Future studies targeting these important questions will help to differentiate between learning, regulation mechanisms and regulation effects. There are also other factors that might influence neurofeedback performance (e.g., intelligence, personality traits). Therefore, future studies with extensive behavioral meta-data are needed to identify all main behavioral influences on neurofeedback.

In addition, it should be noted that this study was conducted on healthy subjects as a first step towards the use of neurofeedback in the field of pain. An external pain stimulus was used as a model for pathologic pain. However, pain processing might differ slightly in chronic pain patients, which should be assessed in a future study. Based on our findings, we hypothesize that behavioral therapy aiming at a more active pain coping could increase neurofeedback efficacy in these subjects as well.

## Conclusion

Our results demonstrate that neurofeedback success is associated with individual behavioral traits. Individual coping styles for pain are associated with pain perception and brain activation during rt-fMRI neurofeedback and the regulation success. Future neurofeedback studies should assess which regulation strategies are best suited for subjects with poor pain coping mechanisms to increase their regulation success and therefore to increase the benefit of neurofeedback.

## Electronic supplementary material

Below is the link to the electronic supplementary material.

ESM 1 (DOCX 43 kb)

ESM 2 (DOCX 11 kb)

## Abbreviations

ACC: Anterior cingulate cortex
AIC: Anterior insular cortex
BOLD: Blood oxygenation level dependent
CSQ: Coping Strategies Questionnaire
fMRI: Functional magnetic resonance imaging
GLM: General linear model
MPRAGE: Magnetization prepared rapid gradient echo
NFB: neurofeedback
NRS: Numeric rating scale
PC: Principle component
PCA: Principle component analysis
PIC: Posterior insular cortex
rt-fMRI: Real-time fMRI
