# Supplementary material for: Active pain coping is associated with the response in real-time fMRI neurofeedback during pain
Source: Brain Imaging Behav. 2016 Apr 12;11(3):712–21. doi: 10.1007/s11682-016-9547-0 (PMC5486591; doi:10.1007/s11682-016-9547-0)
Supplement: Supplementary file 1 — (DOCX 43 kb) [file 11682_2016_9547_MOESM1_ESM.docx]

**Supplementary Table 1** Neurofeedback Strategies

| **Subject** | **Strategy** | **Target Region** |
| --- | --- | --- |
| 1 | Concentrate on cursor, not pain | AIC |
| 2 | Imagination of movement |
| 3 | Mental singing |
| 4 | Increase muscle tension |
| 5 | Fixate a line |
| 6 | Repeat thoughts and words |
| 7 | Try to neglect all feelings of the right arm |
| 8 | Try to move line downward |
| 9 | Calculation |
| 10 | Breathing control |
| 11 | Calculation |
| 12 | Try to conquer pain |
| 13 | - |
| 14 | Progressive muscle relaxation |
| 15 | Try to influence line | ACC |
| 16 | Try to perceive pain as less strong |
| 17 | Visualize pushing pain away |
| 18 | Remember poems |
| 19 | Relax through breathing |
| 20 | Attention on breathing |
| 21 | Repeat certain thoughts |
| 22 | - |
| 23 | Breathing control |
| 24 | Breathing control |
| 25 | - |
| 26 | Perceive heat as cold |
| 27 | Concentrate on line |
| 28 | Draw line with the eyes |
