# Supplementary material for: Active pain coping is associated with the response in real-time fMRI neurofeedback during pain
Source: Brain Imaging Behav. 2016 Apr 12;11(3):712–21. doi: 10.1007/s11682-016-9547-0 (PMC5486591; doi:10.1007/s11682-016-9547-0)
Supplement: Supplementary file 2 — (DOCX 11 kb) [file 11682_2016_9547_MOESM2_ESM.docx]

**Supplementary Table 2** Significant clusters for correlation of BOLD responses during neurofeedback with PC 1 of the CSQ scores (figure 6).

| Cluster Index | Voxels | Maximum z-score | MNI coordinates of local maximum | | | MNI coordinates of center of gravity | | |
| --- | --- | --- | --- | --- | --- | --- | --- | --- |
|  |  |  | X | Y | Z | X | Y | Z |
| 2 | 4189 | 5.03 | -26 | -76 | -2 | -8 | -48 | 0 |
| 1 | 2127 | 4.77 | 22 | 26 | 6 | -2 | 36 | 8 |
